# Lipidomics reveals serum lipid metabolism disorders in CTD-induced liver injury

**DOI:** 10.1186/s40360-024-00732-y

**Published:** 2024-01-15

**Authors:** Shan Li, Xiaotong Duan, Yixin Zhang, Cancan Zhao, Ming Yu, Xiaofei Li, Xiaomei Li, Jianyong Zhang

**Affiliations:** 1 School of Basic Medicine, Zunyi Medical University, Zunyi, 563000 Guizhou China; 2 School of Pharmacy and Key Laboratory of Basic Pharmacology Ministry Education and Joint International Research Laboratory of Ethnomedicine Ministry of Education, Zunyi Medical University, Zunyi, 563000 Guizhou China; 3 Cancer Research Laboratory, The Affiliated Hospital of Zunyi Medical University, Zunyi, 563003 Guizhou China

**Keywords:** CTD, Lipidomics, Hepatotoxicity, Lipid metabolisim, Glycerophospholipid metabolic pathway

## Abstract

**Background:**

Cantharidin (CTD), the main toxic component of *Mylabris*, has been extensively used for tumor treatment in recent years. CTD-induced liver toxicity has attracted significant interest in clinic.

**Methods:**

In this study, biochemical parameters and liver pathological changes were analyzed after CTD was administered to mice by gavage. Subsequently, a lipidomic approach was used to investigate serum lipid metabolism disorders, and the mechanism underlying CTD-induced liver injury in mice was explored.

**Results:**

The results showed that the levels of TC and LDL-C were significantly increased after CTD intervention. Besides, pathological results showed inflammatory cell infiltration and hepatocyte necrosis in the liver. Furthermore, lipidomics found that a total of 18 lipid metabolites were increased and 40 were decreased, including LPC(20:4), LPC(20:3), PC(22:6e/2:0), PE(14:0e/21:2), PC(18:2e/22:6), glycerophospholipids, CE(16:0), CE(18:0) Cholesterol esters and TAG(12:0/12:0/22:3), TAG(16:1/16:2/20:4), TAG(18:1/18:1/20:0), TAG(16:2/18:2/18:2), TAG(18:0/18:0/20:0), TAG(13:1/19:0/19:0) glycerolipids. Metabolic pathway analysis found that glycerophospholipid, glycerol ester and glycosylphosphatidylinositol (GPI)-anchored biosynthetic metabolic pathways were dysregulated and the increase in PE caused by glycophoric metabololism and GPI may be the source of lipid metabolism disorders caused by CTD. Overall, the present study provided new insights into the mechanism of CTD-induced liver injury and increased drug safety during clinical application.

## Introduction

*Mylabris* is the dried body of *Mylabris phalerata Pallas* used for treating carbuncles, ringworm, scrofula and obstruction according to the pharmacopoeia (2020 edition) [1, 2]. In recent years, *Mylabris* has been used for the treatment of liver cancer and ovarian cancer during clinical practice [3–5]. Cantharidin (CTD) is reported the main active substance of *Mylabris* and plays an important role in the treatment of liver cancer. Nonetheless, CTD also has strong hepatotoxicity, which greatly limits clinical application. Therefore, it is necessary to explore the toxicological mechanism of CTD to reduce the occurrence of toxicity.

Studies have substantiated that CTD could induce hepatocyte necrosis, significantly increasing serum alanine aminotransferase (AST) and inducing pathological changes. The hepatotoxicity mechanism of CTD has been associated with inflammation, oxidative stress and endoplasmic reticulum expansion [6]. Meanwhile, it has been reported that CTD could cause hepatotoxicity by increasing the level of glutathione and downregulating the level of 3-sulfalanine in mice, resulting in disturbed glutathione and taurine metabolism [7]. Furthermore, metabolomics studies found that CTD could disrupt triglyceride (TAG) and acylcarnitine metabolism in LO2 hepatocytes, resulting in hepatotoxicity by disrupting lipid metabolism [8]. These studies suggested that CTD could cause hepatotoxicity by affecting the levels of lipid metabolites in mice. However, the mechanisms by which CTD affects lipid metabolism disorders need to be further elucidated.

Lipids are essential metabolites with many key cellular functions acting as cellular barriers and participating in energy metabolism and cell signaling [9]. Moreover, lipid synthesis, metabolism and accumulation are critical for liver homeostasis. Current evidence suggests that imbalances in lipid metabolism play an important role in liver injury [10]. The accumulation of adipose tissue in the liver of mice could disrupt the homeostasis of hepatic metabolism [11]. Some studies have shown that hepatic injury caused by *Polygonum multiflorum*, Phytolacca acinosa Roxb and Zhi-Zi-Hou-Po decoction were associated with lipid metabolism disorders [12–14]. Moreover, untargeted metabolomics studies have found that CTD could affect lipid metabolism and induce liver injury by altering the levels of metabolites such as PE (20:5(5Z,8Z,11Z,14Z,17Z)/14:1(9Z)) and oleic acid in mice [15]. However, untargeted metabolomics only covers a few lipids, which is not specific for lipid metabolite detection, with limited precision and accuracy for lipid metabolite detection [16]. Lipidomics, which can target large amounts of lipids, is more effective, sensitive and more targeted compared to untargeted metabolomics [17]. Moreover, lipidomics has been widely used in various aspects of lipid biochemistry, clinical biomarker discovery and disease diagnosis. For example, a lipidomic study found that serum levels of Lyso-PCs, Cers and SM were significantly decreased in patients with valproic acid-induced hepatotoxicity, while the levels of TAGs with higher total carbon numbers were significantly increased [18].

In the present study, we used biochemical parameters and pathological staining to observe the disturbance of liver lipid metabolism in mice induced by CTD, and further lipidomics analysis was conducted to study the toxic effects and mechanism of CTD on lipid metabolites in mice to prevent CTD-induced hepatotoxicity and expand the clinical application scope of CTD.

## Materials and methods

### Reagent

CTD (purity above 99%, Sigma, USA); Sodium carboxymethyl cellulose (Solarbio, China); PBS (Solarbio, China); Aspartate aminotransferase kit (AST), Alanine transferase kit (ALT), Alkaline phosphatase kit (ALP), Lactate dehydrogenase kit (LDH), Triglyceride kit (TG), Cholesterol kit (TC), High-density lipoprotein kit (HDL-C) and Low-density lipoprotein kit (LDL-C) Boxes were purchased from Nanjing Jiancheng Co., Ltd.

### Animal experimental design

**Fig. 1:**
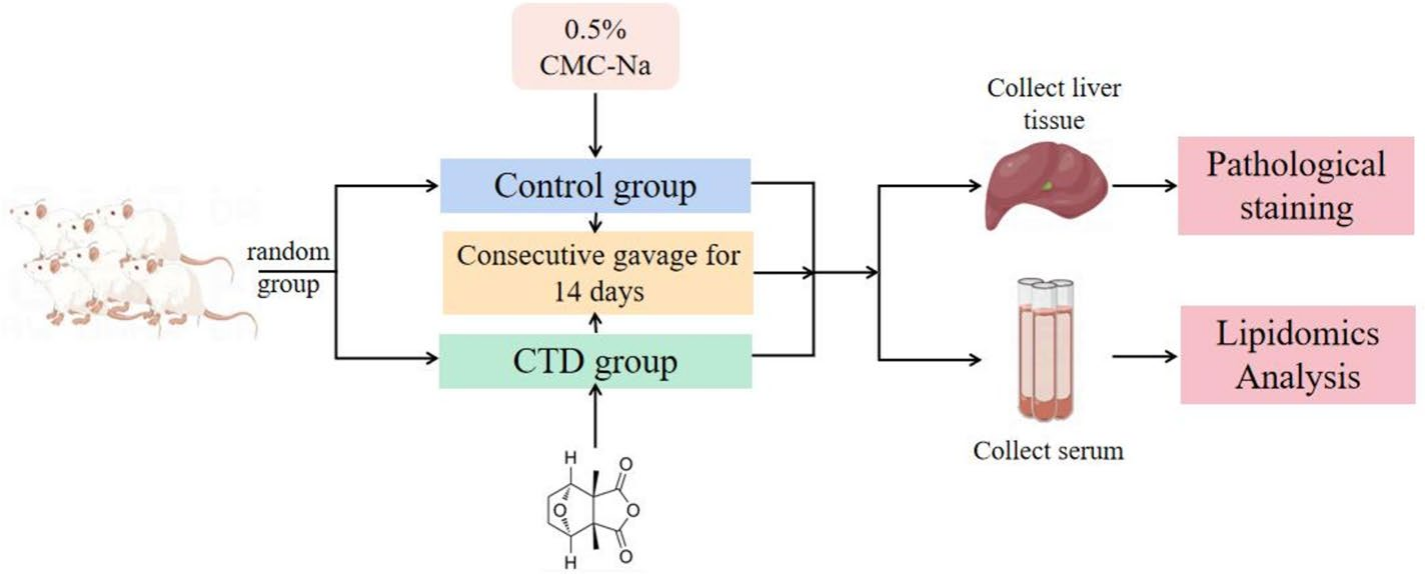
Experimental process chart

Male Balb/c mice were purchased from Chongqing Tengxin Biotechnology Co., Ltd, (Chongqing, China, SYXK (qian) 2021–0003). After one week of acclimatization, 30 Balb/c mice were randomly divided into a control group, which received a concentration of 0.5% carboxymethylcellulose solution (CMC-Na) daily by oral gavage and CTD group, which was administered 1.5 mg/kg of CTD daily by oral gavage. Both groups were treated for 14 days, with free access to water and food during the experimental period. After 14 days of CTD oral gavage, the mice were fasted for 12 h, anesthetized mice with a concentration of 2% -2.5% isoflurane, serum was collected from the mice by eyeball removal, the liver of mice was harvested by dissection and weighed. One part of liver tissue was taken, washed with PBS, and placed in a centrifuge tube with tissue fixative for HE and Oil Red O staining, respectively. The liver index was calculated according to the formula: liver index % = liver weight (g) / body weight (g) × 100%. The overall experimental process is shown in the Fig. 1. The experimental protocol was carried out according to ARRIVE guidelines (serial: 18–2019) as approved by the Animal ethics committee of Zunyi Medical University with an ethical approval number (No.: ZMU21-2105–013).

### Histological analyses

HE staining was used to observe histopathological changes in the liver of mice after CTD intervention. Liver tissue was fixed in 4% paraformaldehyde for 48 h and then the soaked liver tissue was dehydrated in an ethanol series, paraffin-embedded and cut into 5-μm thick sections. Finally, the sections were stained with HE and placed under a microscope for observation.

Oil Red O staining was used to observe the accumulation of lipid droplets in liver tissue. A portion of the 4% paraformaldehyde-fixed liver tissue was frozen and 5 μm-thick frozen liver sections were obtained. Liver sections were stained with 0.5% Oil Red O containing 60% isopropanol for 30 min, further washed with water to remove Oil Red O stain, then incubated with hematoxylin stain for 10 min at room temperature, and washed with water for 5 min. Finally, the liver sections were observed under a Limbus BX41 microscope.

### Lipidomics analysis

#### Extraction of serum lipids

The serum samples were thawed on ice at 4 ℃, vortexed for 30 s, and 100 μL of supernatant was added to a 1.5 ml EP tube with a pipette, 300 μL of extraction solution (methanol:water = 4:1, IS = 10 μg/mL) was added to the supernatant. The supernatant was then vortexed for 30 s, sonicated in an ice-water bath for 5 min, further placed in a -40°C refrigerator for 1 h, and centrifuged at 12,000 rpm for 15 min at 4°C. Finally, the supernatant was pipetted into an injection vial with an internal cannula for analysis and 20ul of supernatant from all samples was mixed into the QC sample.

#### Analysis by UHPLC-QE-MS

LC–MS/MS analysis was used to analyze serum lipids using a UHPLC system (1290, Agilent Technologies), and the target lipids compounds were separated by Phenomen Kinetex C18 liquid chromatography column (2.1 × 100 mm, 1.7 μm). Phase A of liquid chromatography consisted of 40% water and 60% acetonitrile solution, which contained 10 mmol/L ammonium formate, with 50 mL of 10 mmol/L solution added to every 1000 mL aqueous ammonium formate solution. Phase B consisted of 10% acetonitrile and 90% isopropanol solution. The chromatography process was as follows: Gradient elution: 0 ~ 1.0 min, 40% B; 1.0 ~ 12.0 min, 40% ~ 100% B; 12.0 ~ 13.5 min, 100% B; 13.5 ~ 13.7 min, 100% ~ 40% B; 13.7 ~ 18.0 min, 40% B. Mobile phase flow rate: 0.3 mL/min, column temperature: 55 °C, sample tray temperature: 4°C, injection volume: 2 μL. The detailed parameters of the mass spectrometer were set as follows: Sheath gas flow rate: 30 Arb, Auxiliary gas flow rate: 10 Arb, Capillary temperature: 320°C ( +) or 300°C (-), Full ms resolution: 70000, MS/MS resolution: 17500, Collision energy: 15/30/45 in NCE mode, Spray voltage: 5 kV ( +) or -4.5 kV (-).

#### Multivariate statistical analysis

LipidSearch software version 4.1 (Thermo Scientific) was used for peak identification, extraction, alignment and quantification. For the data extraction by LipidSearch, lipid molecules with missing values > 50% were deleted, and the data were normalized to obtain the total peak area. The software SIMCA-P 14.1 (Umetrics, Umea, Sweden) was used for pattern recognition, and the data were preprocessed by Pareto-scaling for multidimensional statistical analysis, including principal component analysis (PCA) and least squares discriminant analysis (PLS-DA).

#### Differential lipid metabolite screening

Lipid differential metabolites were screened for based on the criteria of *P* < 0.05 and FC < 0.05 or FC > 1.5. In addition, the online databases including KEGG (http://www.genome.jp/kegg/) and MetaboAnalyst (http://www.metaboanalyst.ca/) were used for pathway enrichment analysis.

### Data analysis

All experimental data were expressed as means ± standard error (x ± SEM). Data were subjected to one-way ANOVA using GraphPad Prism 8 (GraphPad Software, San Diego, CA), with* p* < 0.05 indicating significance and *p* < 0.01 indicating significant differences.

## Results

### Changes in body weight and liver index of mice

**Fig. 2:**
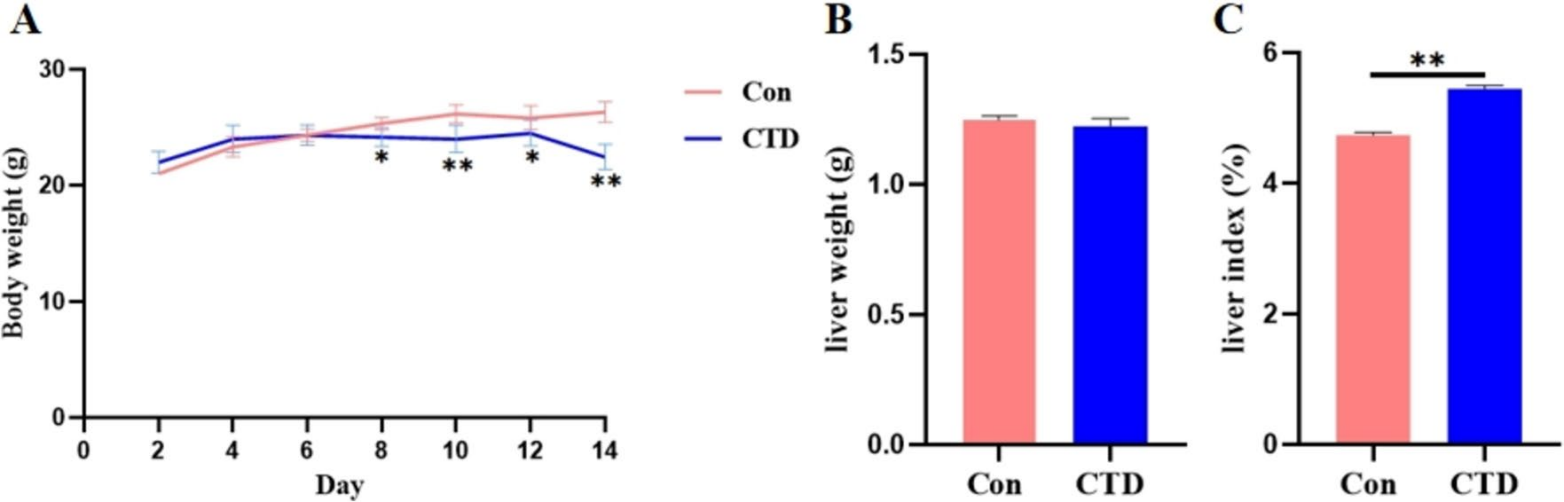
The body weight (**A**), liver weight (**B**) and liver index (**C**) changes after CTD in mice, (‾x ± SEM, *n* = 6). Compared with the Con group: *, *P* < 0.05; **, *P* < 0.01

Organ coefficient, as an indicator of toxicology, was often used to detect drug toxicity. As shown in Fig. 2, compared with the control group, as the number of days of CTD oral gavage increased, the body weight of the CTD group mice gradually decreased (Fig. 2A, *P*<0.05 or *P*<0.01) and the liver index increased (Fig. 2C, *P*<0.01). But, there was no significant change in liver weight in mice (Fig. 2B). Above results suggested that CTD may disturb the metabolism in mice.

### Serum biochemical indicator detection

**Fig. 3:**
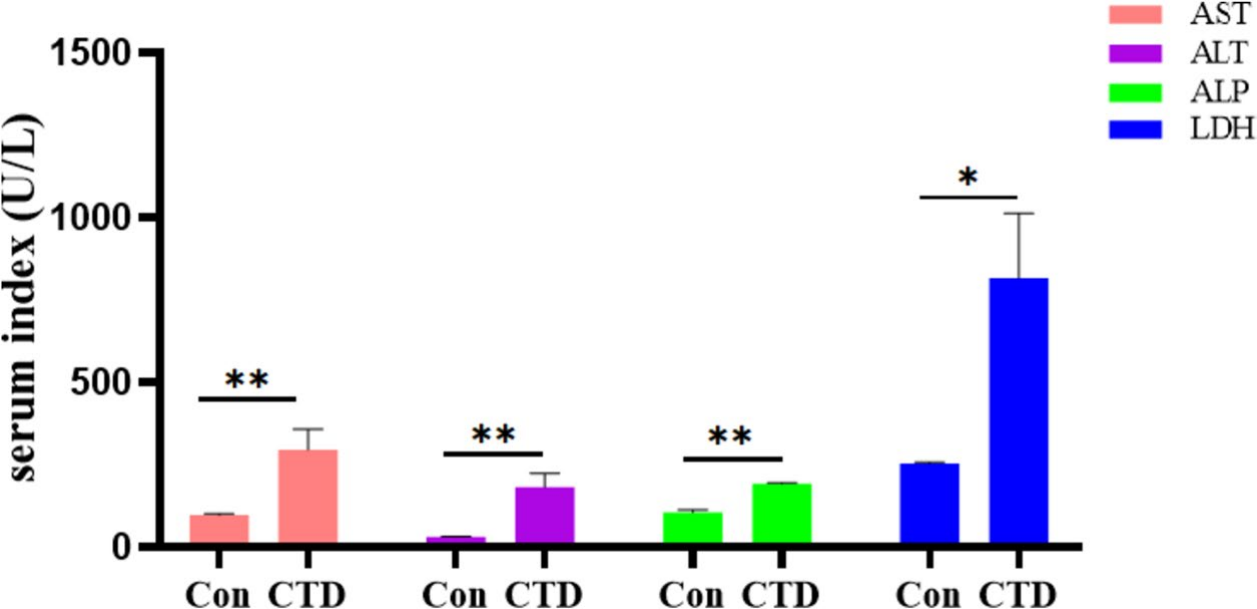
The effect of CTD on the levels of serum liver function biochemical indexes. (‾x ± SEM, *n* = 6). Compared with the Con group: *, *P* < 0.05; **, *P* < 0.01

The effect of CTD on the levels of serum liver function biochemical indexes was shown in Fig. 3. Compared with the Con group, the serum levels of AST, ALT, and ALP in the CTD group were significantly increased (*P* < 0.01), and the level of LDH was increased (*P* < 0.05), suggesting that CTD could cause liver damage in mice.

**Fig. 4:**
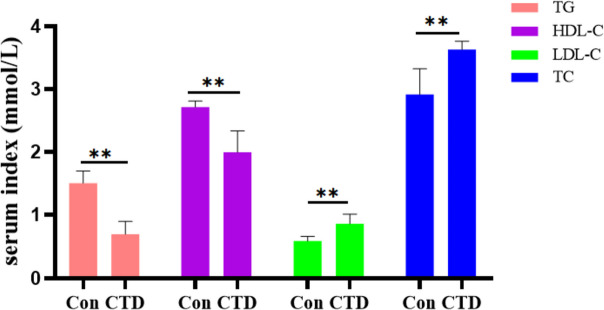
The effect of CTD on serum lipid biochemical indicators(‾x ± SEM, *n* = 6). Compared with the Con group, *, *P* < 0.05; **, *P* < 0.01

Serum biochemical indicators of lipid metabolism were also determined, as shown in Fig. 4. The levels of TC and HDL-C in CTD group were significantly decreased (*P* < 0.01) and the levels of TG and LDL-C were significantly increased (*P* < 0.01) compared with the Con group, suggesting that CTD-induced liver injury may be related to lipid metabolism disorders in vivo.

### Histopathological examination

**Fig. 5:**
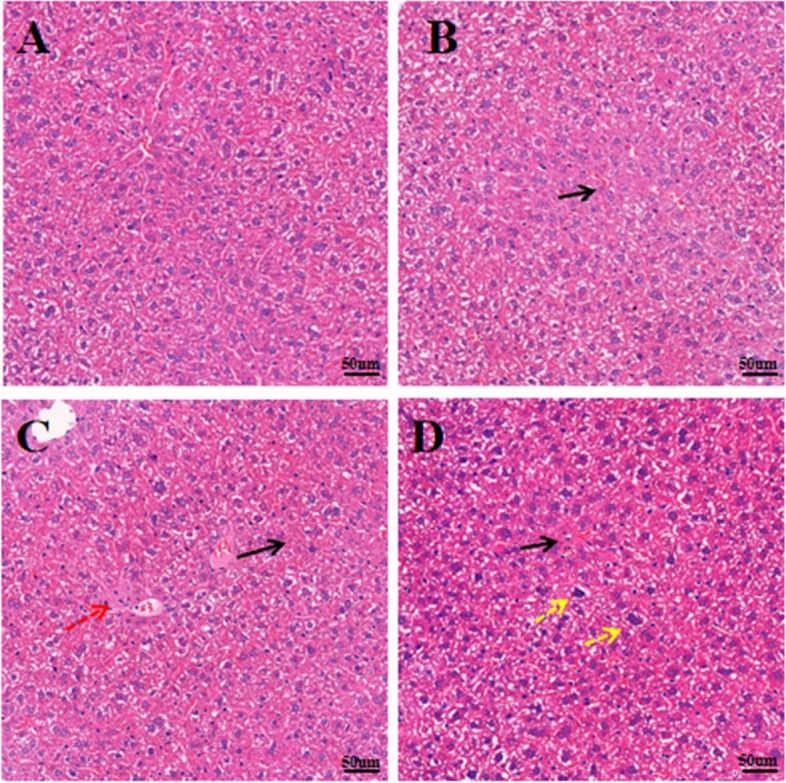
HE staining pathological results of mice liver tissue, Scale bar: 50 µm. **A** represents the Con group, and **B**, **C**, and **D** represent the CTD groups. Black arrows indicate hepatocyte necrosis; red arrows indicate inflammatory cell infiltration; yellow arrows indicate hepatocyte swelling

The liver tissue of mice subsequently underwent HE staining (Fig. 5). Histopathological examination showed that the liver tissue of the Con group exhibited a normal hepatocyte structure and no pathological changes (Fig. 5A), while the liver of mice in the CTD group showed hepatocyte swelling, increased mitotic figures, inflammatory cell infiltration and hepatocyte necrosis (5B, C, D).

**Fig. 6:**
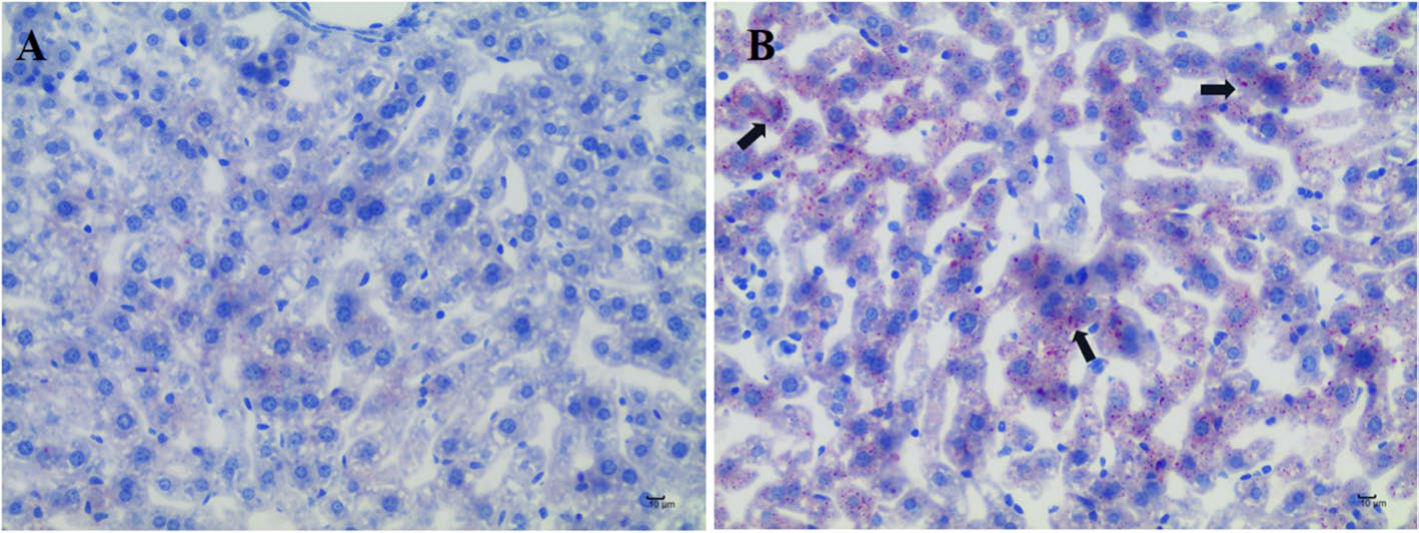
Oil red O staining results of mice liver tissue, Scale bar: 10 µm. **A** is the Con group and **B** is the CTD group. Lipids in red and hepatocytes in blue. Black arrows represent lipid accumulation

In order to further study the effect of CTD on lipid accumulation in the liver of mice, the liver tissue of mice was stained with Oil Red O (Fig. 6). Compared with the Con group, the liver tissue of the mice in the CTD group showed excessive lipid accumulation and slight steatosis.

### Lipidomics analysis of CTD-induced liver injury in mice

#### QC sample detection and system stability analysis

**Fig. 7:**
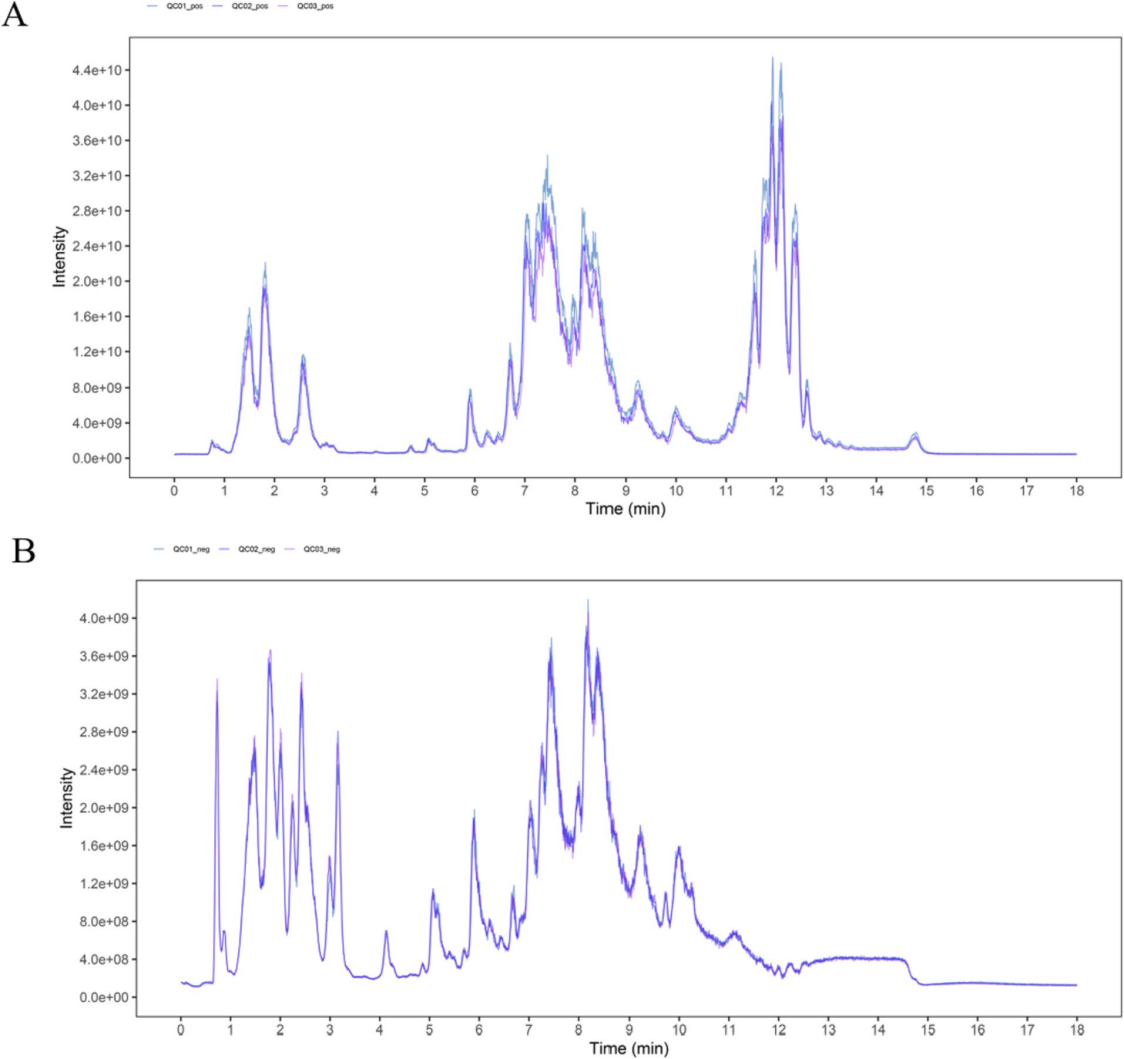
The TIC overlaps of QC samples in positive ion mode (POS, **A**) and negative ion mode (NEG, **B**). X axis and Y axis stand for time (min) and intensity

In this study, 9473 peaks were detected in positive ion mode and 8773 peaks were detected in negative ion mode by performing relative standard deviation (RSD), coefficient of variation (CV) and normalized filtering noise reduction processing on the raw data. The total ionization chromatogram (TIC) of the six QC samples was used to evaluate the repeatability and stability of the system. The results showed good retention time and peak area overlap for the QC samples, indicating that the instrument was stable enough for subsequent experiments (Fig. 7).

#### Multivariate statistical analysis

**Fig. 8:**
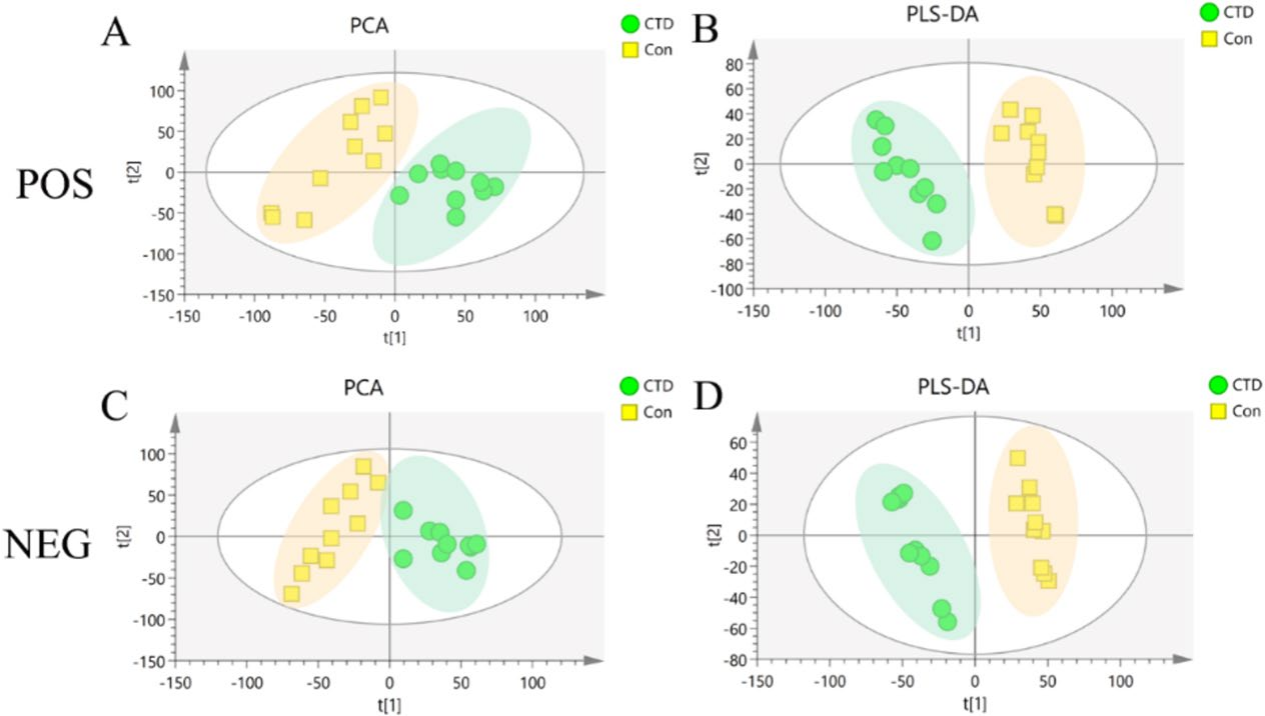
Principal component analysis (PCA) and Partial least-squares discriminant analysis (Partial least-squares discriminant analysis, PLS-DA)) in POS (**A**, **B**) and NEG (**C**, **D**) of the Con group and the CTD group score map

As shown in Fig. 8A and C, PCA was used to analyze changes in the lipid metabolism profiles in mice after CTD intervention. Compared with the Con group, the metabolic profiles of the mice in the CTD group were significantly changed in both POS and NEG with no outliers. An in-depth analysis was performed using PLS-DA to further explore the metabolic differences caused by CTD. The results showed that in both two modes, metabolic profiles in the CTD group were significantly separated from the Con group, indicating differences in the metabolite levels between the two groups (Fig. 8B and D). The modeling data of different models are shown in Table 1.

**Table 1:** Modeling parameters of different models of serum metabolites

Model	Mode	Type	A	N	R^2^X	R^2^Y	Q^2^
Model 1	POS	PCA	5	20	0.591	-	0.126
Model 2	NEG	PCA	5	20	0.518	-	0.043
Model 3	POS	PLS-DA	5	20	0.526	1	0.963
Model 4	NEG	PLS-DA	5	20	0.453	1	0.968
Model 5	POS	OPLS-DA	1 + 3 + 0	20	0.465	0.998	0.881
Model 6	NEG	OPLS-DA	1 + 3 + 0	20	0.41	0.1	0.867

**Fig. 9:**
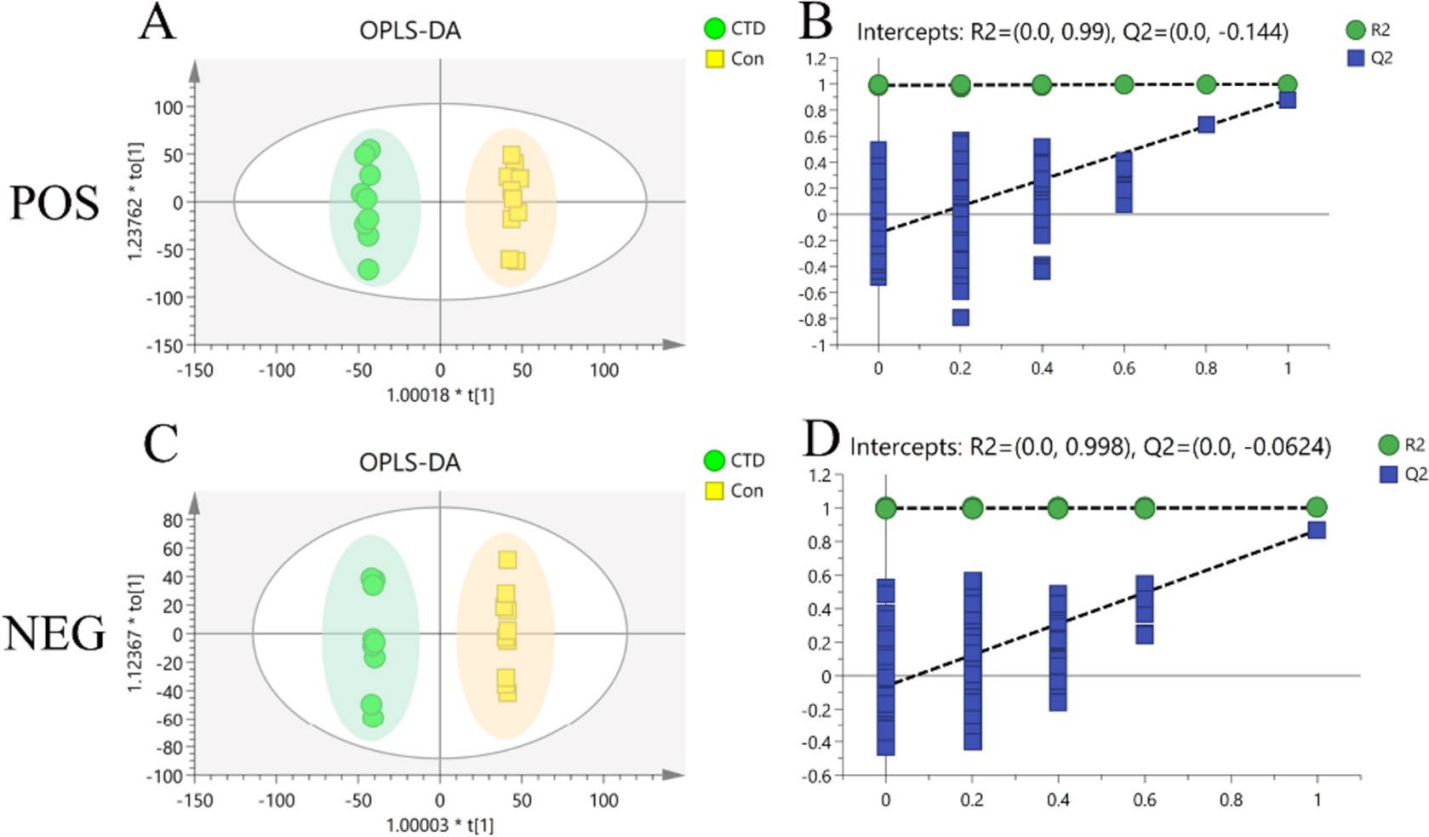
Orthogonal partial least-squares discriminant analysis (OPLS-DA) scores of the Con group and the CTD group in POS (**A**, **B**) and NEG (**C**, **D**) and permutation test plot (200 times)

To further elucidate the differential variables between the Con group and the CTD group, an OPLS-DA analysis was performed for supervised pattern recognition. Simultaneously, a permutation test was performed on the metabolic data, repeated 200 times, and the results showed that the data were not overfitted, as shown in Fig. 9. The OPLS-DA results showed that the two groups of samples were completely distinct.

#### Screening of differential lipid metabolites

**Fig. 10:**
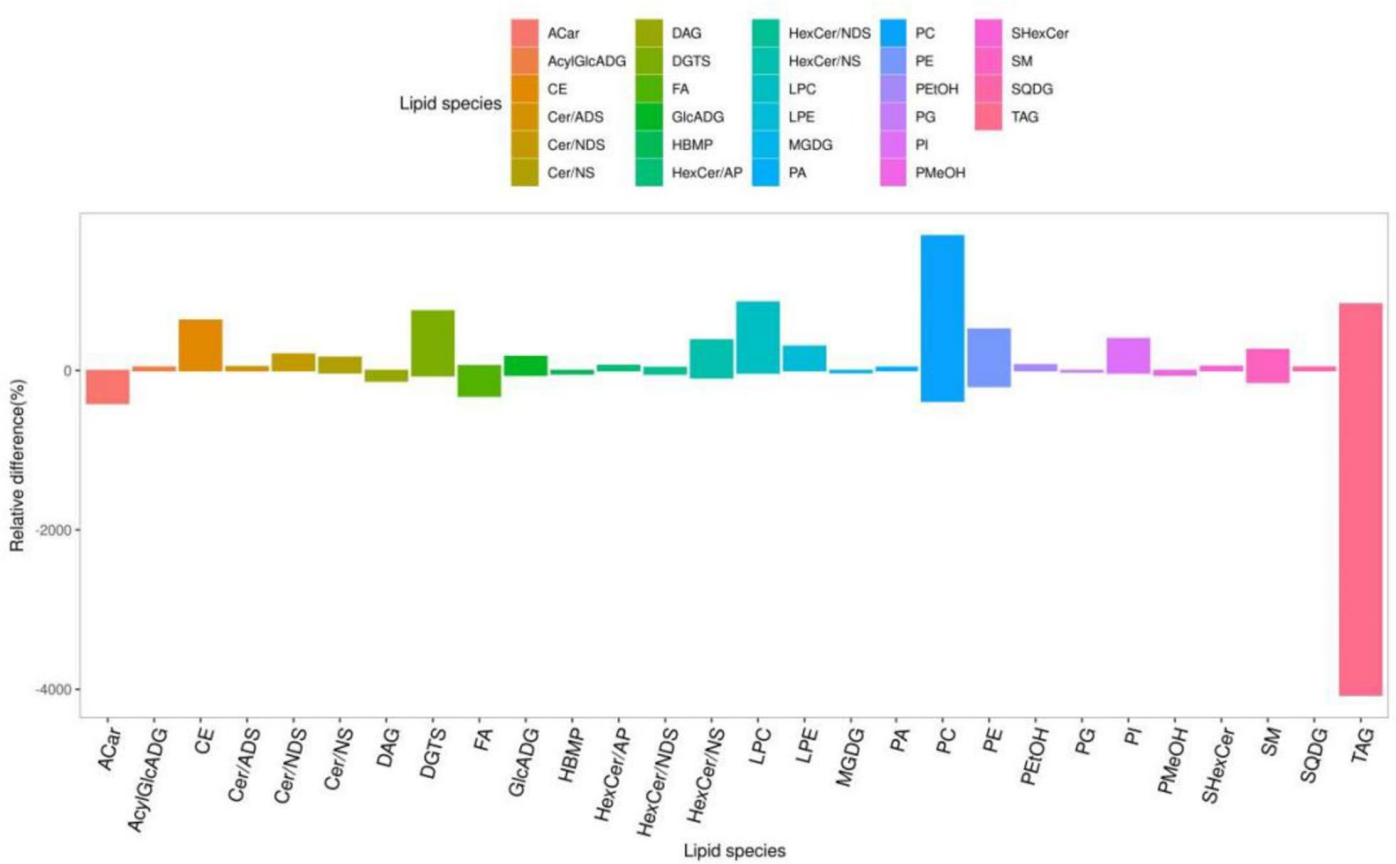
Effects of CTD on serum lipid metabolites

On the basis of OPLS-DA and LIPID MAPS, KEGG and MetaboAnalyst database analysis, 279 potential lipid differential biomarkers were obtained by screening differential lipid metabolites with VIP > 1 and *P* < 0.05. Further, the differential markers were screened with FC > 2 or FC < 0.5, and finally 58 biomarkers were obtained, as shown in Table 2. The potential lipid differential biomarkers mainly included glycerolipids, glycerophospholipids, Sphingomyelin, Polyethylene, fatty acylides and Cholesterol esters, as shown in Fig. 10. The results showed that after 14 days of CTD administration, compared with Con group, PC increased significantly and TAG decreased significantly, suggesting that CTD may induce liver injury mainly by changing the content of glycerophospholipid PC and glycerolipid TAG in mice.

**Table 2:** Serum lipid differential metabolites

No	Name	Inoziation	VIP	*P*-Vaule	Fold Change	KEGG	Type
1	TAG(16:1/16:2/20:4)	ESI +	1.85E + 00	1.15E-03	2.45E-01	-	down
2	TAG(16:1/16:1/18:2)	ESI +	1.80E + 00	7.21E-04	4.42E-01	C00422	down
3	HexCer/NS(d18:1/16:0)	ESI +	1.79E + 00	2.32E-06	2.20E + 00	-	up
4	FA(19:0)	ESI-	1.78E + 00	2.34E-06	4.97E-01	-	down
5	CE(18:0)	ESI +	1.77E + 00	1.00E-03	2.72E + 00	C02530	up
6	PC(19:0/20:4)	ESI-	1.77E + 00	1.04E-06	4.97E-01	-	down
7	FA(22:1)	ESI-	1.76E + 00	3.90E-05	4.57E-01	-	down
8	TAG(14:1/16:1/20:3)	ESI +	1.71E + 00	1.14E-03	4.84E-01	-	down
9	SM(d15:0/26:2)	ESI-	1.68E + 00	3.53E-04	4.39E-01	-	down
10	HexCer/NS(d18:1/24:1)	ESI-	1.67E + 00	4.90E-04	2.59E + 00	-	up
11	FA(20:1)	ESI-	1.67E + 00	2.50E-05	4.94E-01	-	down
12	LPC(20:3)	ESI +	1.65E + 00	1.45E-04	2.08E + 00	C04230	up
13	SM(d14:1/28:2)	ESI-	1.64E + 00	3.96E-04	2.47E + 00	-	up
14	TAG(18:0/18:1/20:0)	ESI +	1.64E + 00	2.98E-04	4.48E-01	C00422	down
15	TAG(14:0/20:4/20:5)	ESI +	1.63E + 00	1.70E-04	4.99E-01	-	down
16	TAG(16:1/16:1/21:2)	ESI +	1.62E + 00	7.23E-04	4.66E-01	-	down
17	PC(22:6e/2:0)	ESI +	1.61E + 00	1.32E-04	2.09E + 00	-	up
18	TAG(12:0/16:0/20:3)	ESI +	1.61E + 00	3.28E-03	4.96E-01	-	down
19	TAG(16:0/20:1/20:1)	ESI +	1.59E + 00	1.69E-03	4.90E-01	C00422	down
20	TAG(18:0/18:0/20:0)	ESI +	1.59E + 00	4.39E-03	4.07E-01	C00422	down
21	DAG(18:2/18:2)	ESI +	1.58E + 00	2.64E-04	4.83E-01	C00165	down
22	TAG(18:1/18:2/22:0)	ESI +	1.58E + 00	4.41E-04	4.62E-01	-	down
23	TAG(18:2/18:2/22:0)	ESI +	1.57E + 00	9.63E-04	4.76E-01	-	down
24	TAG(17:0/17:0/19:0)	ESI +	1.57E + 00	1.36E-03	4.70E-01	-	down
25	TAG(12:0/12:0/22:3)	ESI +	1.57E + 00	1.24E-02	2.28E-01	-	down
26	TAG(18:2/18:2/18:3)	ESI +	1.56E + 00	7.13E-05	4.97E-01	-	down
27	TAG(13:0/13:0/21:4)	ESI +	1.53E + 00	3.39E-05	4.98E-01	-	down
28	TAG(14:0/16:0/22:6)	ESI +	1.48E + 00	3.01E-03	4.62E-01	-	down
29	PI(16:0/20:3)	ESI-	1.47E + 00	1.18E-02	2.24E + 00	C00626	up
30	TAG(18:0/18:0/21:0)	ESI +	1.47E + 00	3.24E-03	4.74E-01	-	down
31	TAG(16:0/20:1/22:1)	ESI +	1.45E + 00	1.43E-03	4.93E-01	-	down
32	TAG(18:1/18:1/20:0)	ESI +	1.44E + 00	1.80E-02	2.75E-01	C00422	down
33	GlcADG(16:0/22:6)	ESI-	1.43E + 00	4.80E-03	2.01E + 00	-	up
34	TAG(16:0/18:2/21:0)	ESI +	1.43E + 00	1.65E-02	4.69E-01	-	down
35	LPC(20:4)	ESI +	1.42E + 00	1.08E-02	2.03E + 00	C04230	up
36	TAG(16:2/18:2/18:2)	ESI +	1.41E + 00	1.68E-04	3.72E-01	-	down
37	DGTS(27:0/22:2)	ESI +	1.39E + 00	3.17E-03	3.76E + 00	-	up
38	TAG(18:1/18:2/21:0)	ESI +	1.39E + 00	3.11E-03	4.90E-01	-	down
39	ACar(18:2)	ESI +	1.39E + 00	1.66E-02	4.48E-01	-	down
40	ACar(14:0)	ESI +	1.38E + 00	2.95E-02	4.39E-01	-	down
41	TAG(20:0/20:1/22:0)	ESI +	1.37E + 00	7.74E-03	4.99E-01	-	down
42	TAG(18:0/18:0/22:0)	ESI +	1.35E + 00	2.42E-02	4.37E-01	-	down
43	DGTS(18:0/27:0)	ESI +	1.34E + 00	2.58E-03	4.20E + 00	-	up
44	TAG(12:3/22:7/22:7)	ESI +	1.33E + 00	8.60E-03	2.05E + 00	-	up
45	TAG(17:3/18:5/19:0)	ESI +	1.33E + 00	5.61E-03	4.82E-01	-	down
46	PC(20:0/27:0)	ESI +	1.32E + 00	5.97E-03	2.98E + 00	-	up
47	TAG(20:1/22:1/22:1)	ESI +	1.29E + 00	7.80E-03	4.94E-01	-	down
48	DGTS(25:0/25:0)	ESI +	1.29E + 00	2.23E-02	3.68E-01	-	down
49	DGTS(27:0/20:1)	ESI +	1.28E + 00	6.81E-03	2.51E + 00	-	up
50	PE(14:0e/21:2)	ESI +	1.24E + 00	2.37E-02	2.96E + 00	-	up
51	ACar(16:1)	ESI +	1.24E + 00	3.09E-02	4.99E-01	-	down
52	TAG(13:1/19:0/19:0)	ESI +	1.24E + 00	7.74E-03	4.26E-01	-	down
53	TAG(12:0/12:0/20:1)	ESI +	1.23E + 00	1.86E-02	4.51E-01	-	down
54	PMeOH(24:4/24:4)	ESI +	1.23E + 00	7.53E-03	4.34E-01	-	down
55	PC(18:0/27:0)	ESI +	1.18E + 00	1.26E-02	2.72E + 00	-	up
56	CE(16:0)	ESI +	1.10E + 00	3.15E-02	2.86E + 00	C11251	up
57	ACar(12:0)	ESI +	1.09E + 00	4.51E-02	4.00E-01	-	down
58	PC(18:2e/22:6)	ESI +	1.07E + 00	1.06E-03	2.04E + 00	-	up

**Fig. 11:**
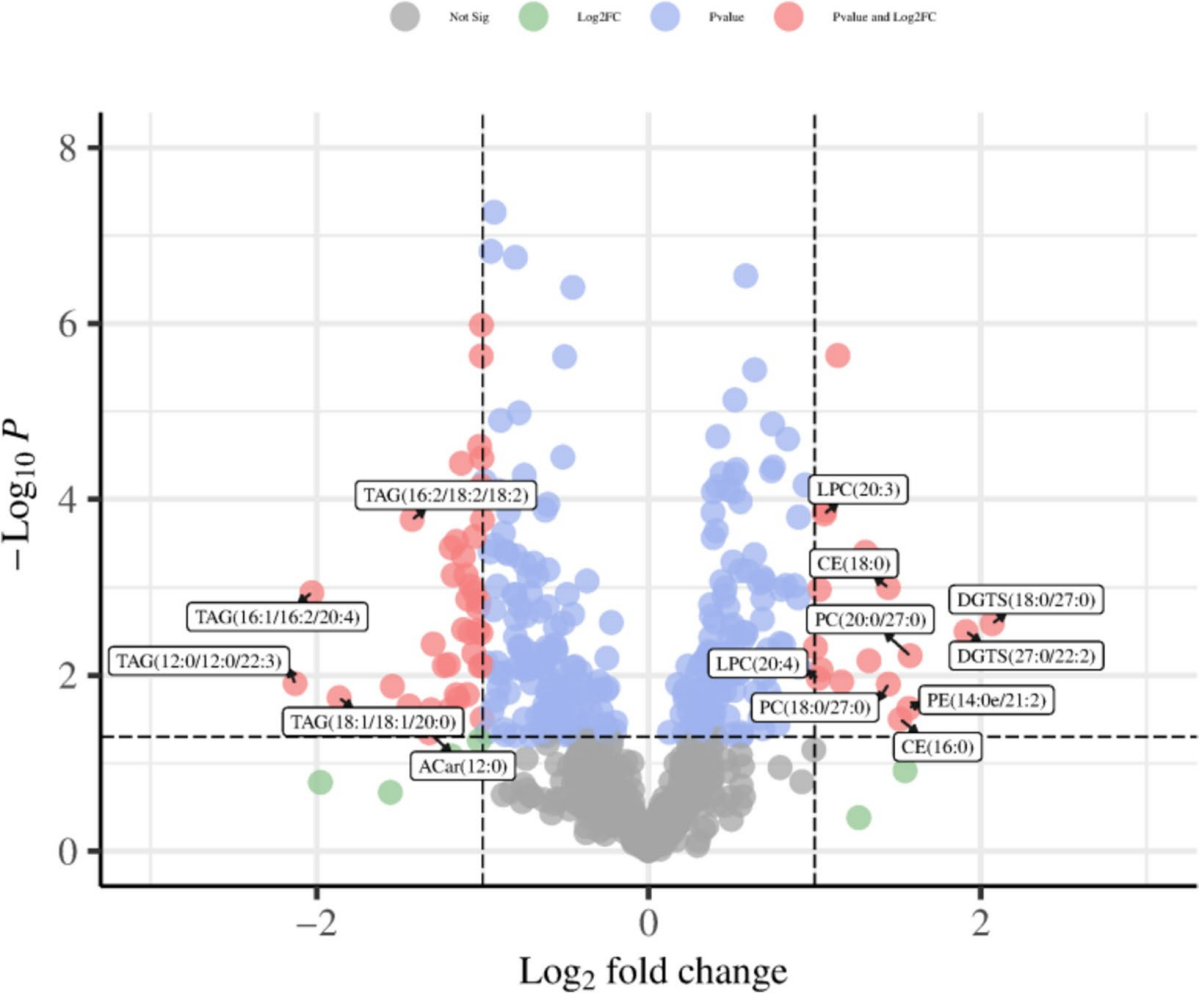
Volcano plot of potential differential metabolites in Con group VS the CTD group. X-axis represents Log2 Fold change, Y-axis represents log10 *P* value

**Fig. 12:**
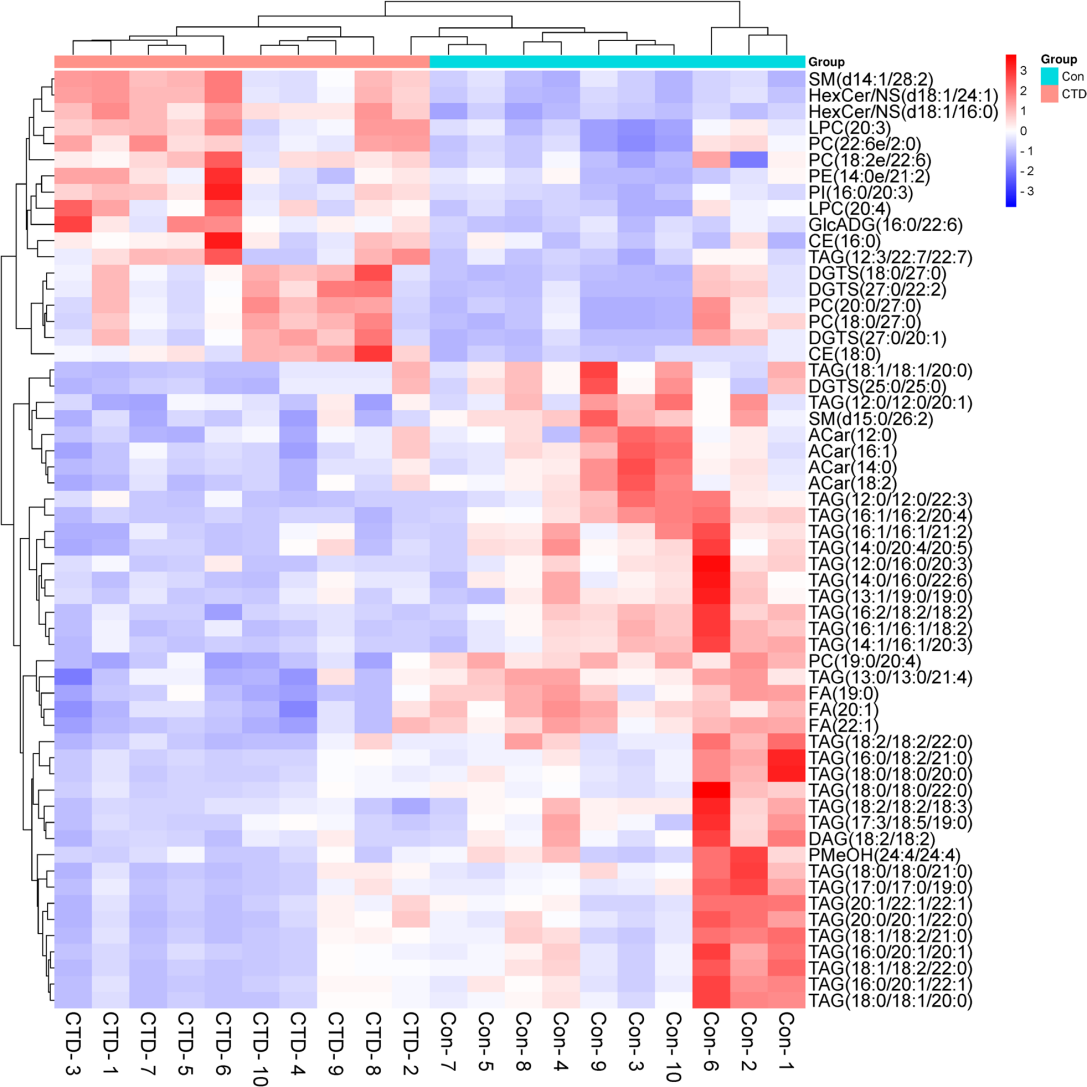
Hierarchical clustering heatmap of serum from the Con and CTD group. The shade of color represents the content of metabolites, red blocks indicate high expression, while blue indicate low expression

To observe the trend of the metabolites between the Con and CTD groups more objectively, the levels of metabolites were further analyzed by volcano plots. The results showed a total of 18 lipid metabolites were increased (red dots) and 40 were decreased (blue dots), as shown in Fig. 11. At the same time, the metabolite levels were visualized in a heatmapto intuitively explore the changes of the overall metabolic profile of the differential metabolites, which were consistent with the results of volcano plot (Fig. 12).

#### Screening of differential lipid biomarkers

**Fig. 13:**
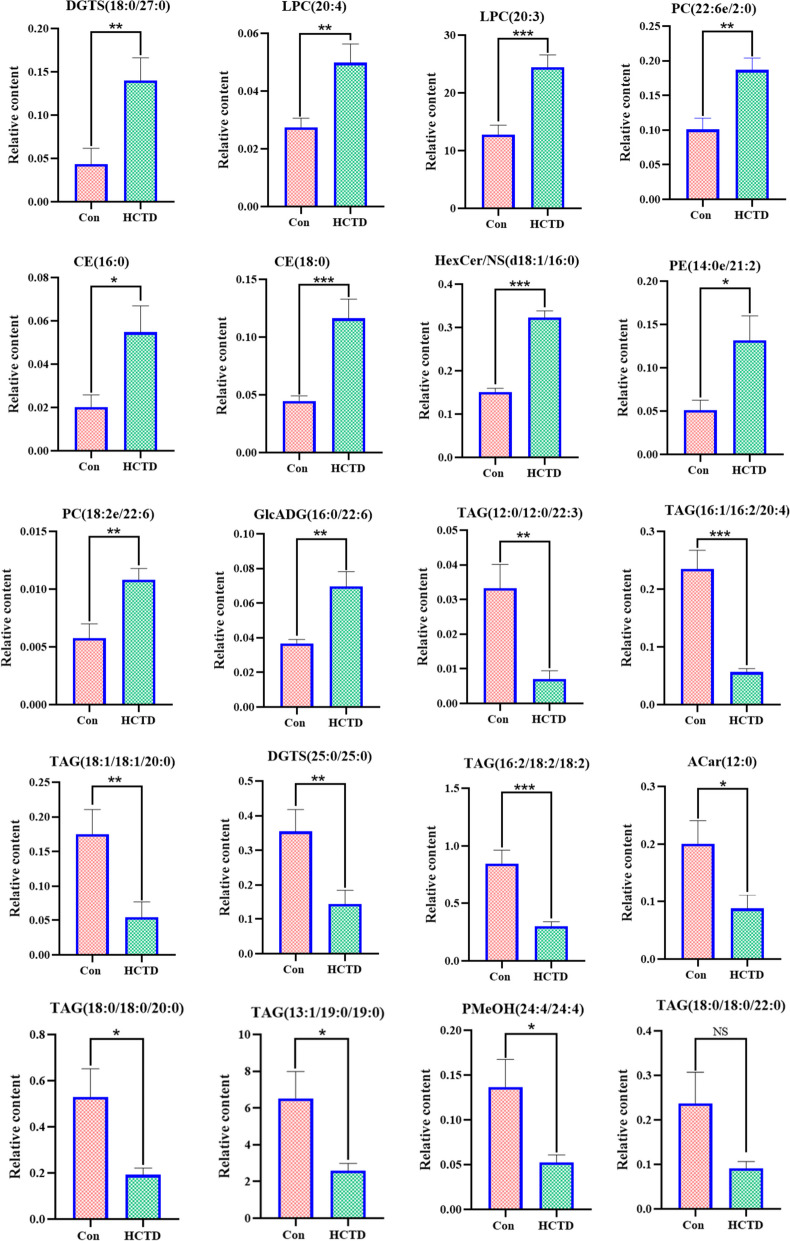
Relative content histogram of top 20 lipid markers

The top ten lipid differential metabolites were further screened in descending order of *P* < 0.05, VIP > 1 and FC > 2 and the top ten lipid differential metabolites were screened in ascending order of P < 0.05, VIP > 1and FC < 0.5, including DGTS(18:0/27:0), LPC(20:4), LPC(20:3), PC(22:6e/2:0), CE(16:0), CE(18:0), HexCer/NS(d18:1/16:0), PE(14:0e/21:2), PC(18:2e/22:6), GlcADG(16:0/22:6), TAG(12:0/12:0/22:3), TAG(16:1/16:2/20:4), TAG(18:1/18:1/20:0), DGTS(25:0/25:0), TAG(16:2/18:2/18:2), ACar(12:0), TAG(18:0/18:0/20:0), TAG(13:1/19:0/19:0), PMeOH(24:4/24:4), TAG(18:0/18:0/22:0). Finally, LPC(20:3), PC(22:6e/2:0), TAG(16:2/18:2/18:2), TAG(13:1/19:0/19:0) were confirmed to be the most likely biomarkers for CTD-induced liver injury in mice according to the content changes of each marker (Fig. 13).

#### Metabolic pathway analysis

**Fig. 14:**
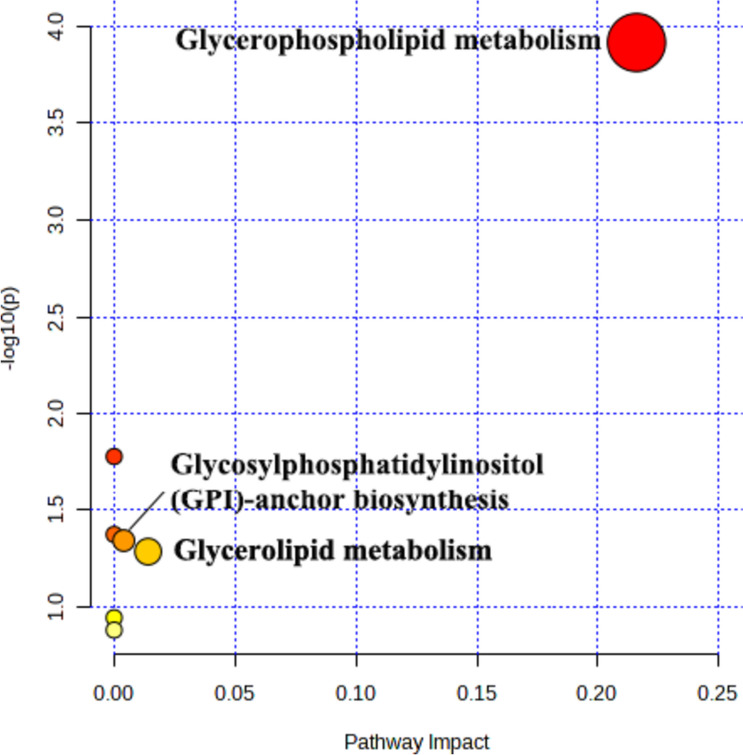
Metabolic pathway analysis of 93 lipid differential metabolites in mice serum. The X-axis represents pathway effects and the Y-axis represents -log10 *P*-values

The disturbed metabolic pathways associated with differential lipid metabolites were analyzed by enrichment analysis. As shown in Fig. 14. 7 pathways were involved in CTD-induced liver injury, including glycerophospholipid, glyceride, linoleic acid, glycosylphosphatidylinositol (GPI)-anchored biosynthesis, peanut tetraenoic acid, alpha-linolenic acid and steroid biosynthesis pathway.

**Fig. 15:**
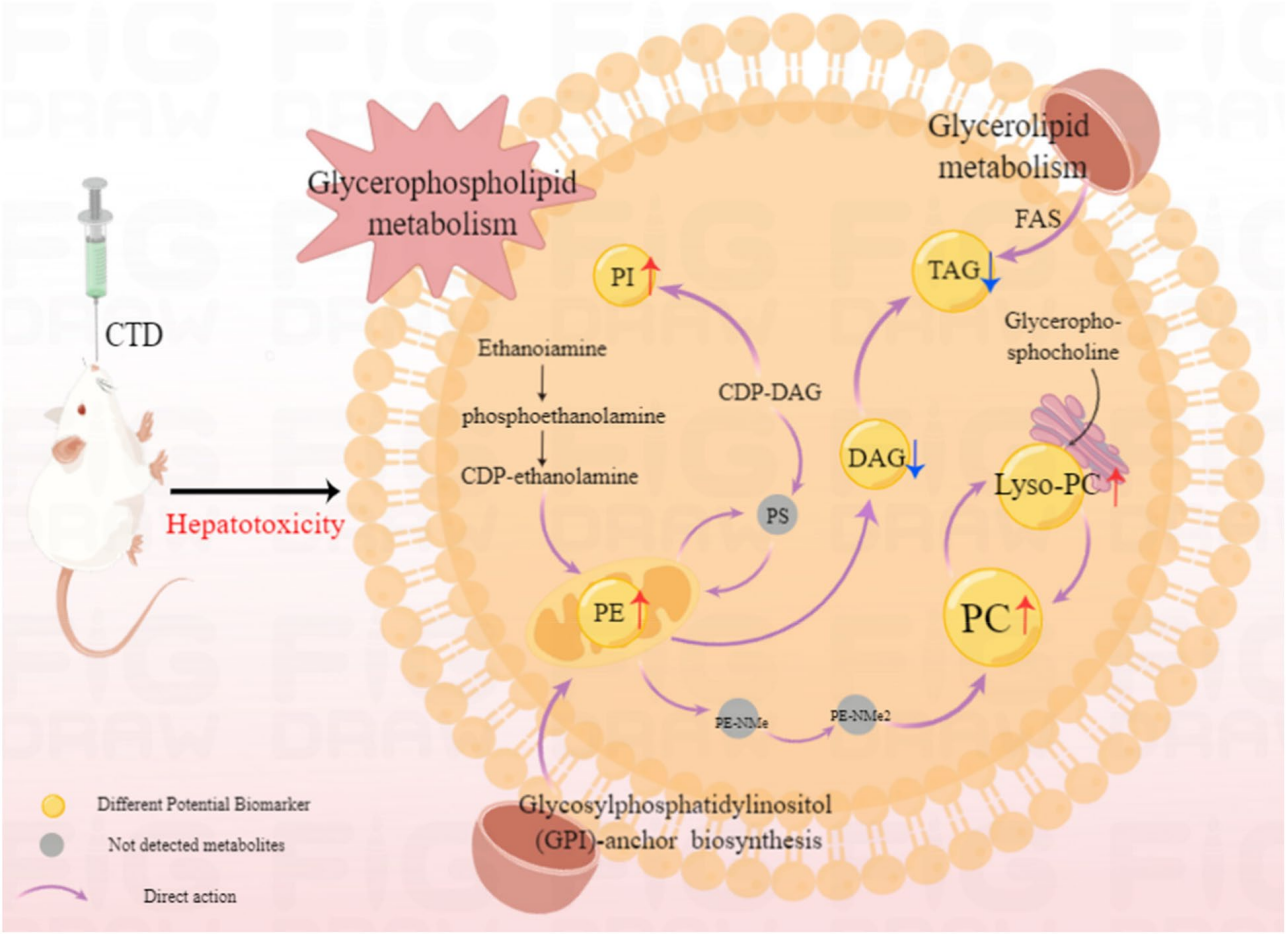
Schematic diagram of CTD regulating lipid metabolism pathway. PE: Phosphatidylethanolamine; PC: Phosphatidylcholine; Lyso-PC: Lysophosphatidylcholine; PI: phosphatidylinositol; PS: Phosphatidylserine; DAG: Diacylglycerol; TAG: Triglycerides; Red upward arrows represent metabolite upregulation; Blue down arrows represent metabolite downregulation

Significant pathways were obtained from the MetaboAnalyst online database, based on the screening criteria *P* < 0.05. The top 3 metabolic pathways were screened, including the glycerophospholipid, the glycerol ester and the glycosylphosphatidylinositol (GPI)-anchored biosynthetic pathways. Specific information on the metabolic pathways is shown in Table 3. The 3 enriched metabolic pathways were all closely related to lipid biosynthesis. Further analysis revealed that PC(22:6e/2:0), PC(18:2e/22:6) and PE (14:0e/21:2) played an important role in the 3 metabolic pathways. KEGG analysis revealed that the 3 metabolic pathways were associated with PE and played a significant role in CTD-induced liver injury, as shown in Fig. 15.

**Table 3:** Information table of metabolic pathways related to lipid differential metabolites

KEGG pathway	*P*-value	Impact	Hits compound
Glycerophospholipid metabolism	5.96E-04	0.2163	PC; PE
Glycerolipid metabolism	7.98E-02	0.0140	TAG
Glycosylphosphatidylinositol (GPI)-anchor biosynthesis	7.02E-02	0.0040	PE

## Discussion

In this study, the traditional pharmacodynamics approach and lipidomics were integrated to demonstrate that CTD administration significantly altered the metabolic profile of serum lipids in mice, triggering a disturbance in lipid metabolism, suggesting that dysregulated lipid metabolism may be the main mechanism of action of CTD-induced liver injury.

### The toxicology effects of CTD-induced liver injury in mice

It is well established that AST, ALT, ALP, and LDH are liver enzymes. During hepatocyte injury, these markers are leaked into the circulatory system due to changes in membrane permeability, resulting in significant increases in the blood. Accordingly, these markers are commonly used during the clinical evaluation of liver injury [19]. Serum levels of TC, TG, LDL-C and HDL-C represent the status of lipids. Dyslipidaemia refers to a range of quantitative and qualitative changes in serum lipids and lipoproteins [20]. In this study, we found that CTD could increase liver function enzymes and the levels of TC and LDL. Liver function and blood lipid indicators indicated potential mice liver injury, leading to abnormal liver function and lipid metabolism disorders, suggesting that CTD-induced liver injury may be related to lipid metabolism disorders in mice.

Furthermore, HE staining results showed local necrosis and other pathological changes in the hepatocytes of the mice, which was consistent with the biochemical indexes. The Oil red O staining results also showed that CTD could cause lipid accumulation in the liver. Taken together, these results further validated that lipid metabolism disorders play an important role in CTD-induced liver injury.

### The effect of CTD on lipid metabolism in mice

Lipidomics enables comprehensive identification and quantification of various lipid molecular species, which broadens our understanding of toxicological effects and the underlying mechanisms [17]. In this study, the UHPLC-QE-MS method was used to investigate disturbances in serum lipid metabolites in mice after CTD-induced liver injury. The results showed that CTD could cause liver lipotoxicity and injury mainly by disrupting glycerophospholipid and glycerol ester metabolites and then acting on pathways such as glycerophospholipid metabolism and glycerol ester metabolism. A total of 58 differential lipid metabolites were disturbed after CTD intervention, mainly including significantly increased PC, Lyso-PC glycerophospholipids, Cholesterol ester (CE) and downregulated TAG glycerides. Notably, further analysis revealed that glycerophospholipid, glyceride, and glycosylphosphatidylinositol (GPI)-anchored biosynthesis pathways are important in the process of CTD-induced liver injury.

#### Glycerophospholipid metabolic pathway

Glycerophospholipids, as thestorage deposits of lipid mediators, are major components of the membrane bio-layer, which are involved in many cellular functions including inflammation response, cell metabolism and signal transduction [21, 22]. Meanwhile, glycerophospholipids are also involved in the formation of mitochondrial membranes. Abnormal lipid metabolism can lead to mitochondrial dysfunction, which in turn leads to lipid deposition in the body and lipid toxicity [23–25]. More interestingly, glycerophospholipids have been reported to exacerbate metabolite disorders and contribute to liver disease by affecting the function of cytochrome P450s and UDP-glucuronosyltransferase [26].

**Fig. 16:**
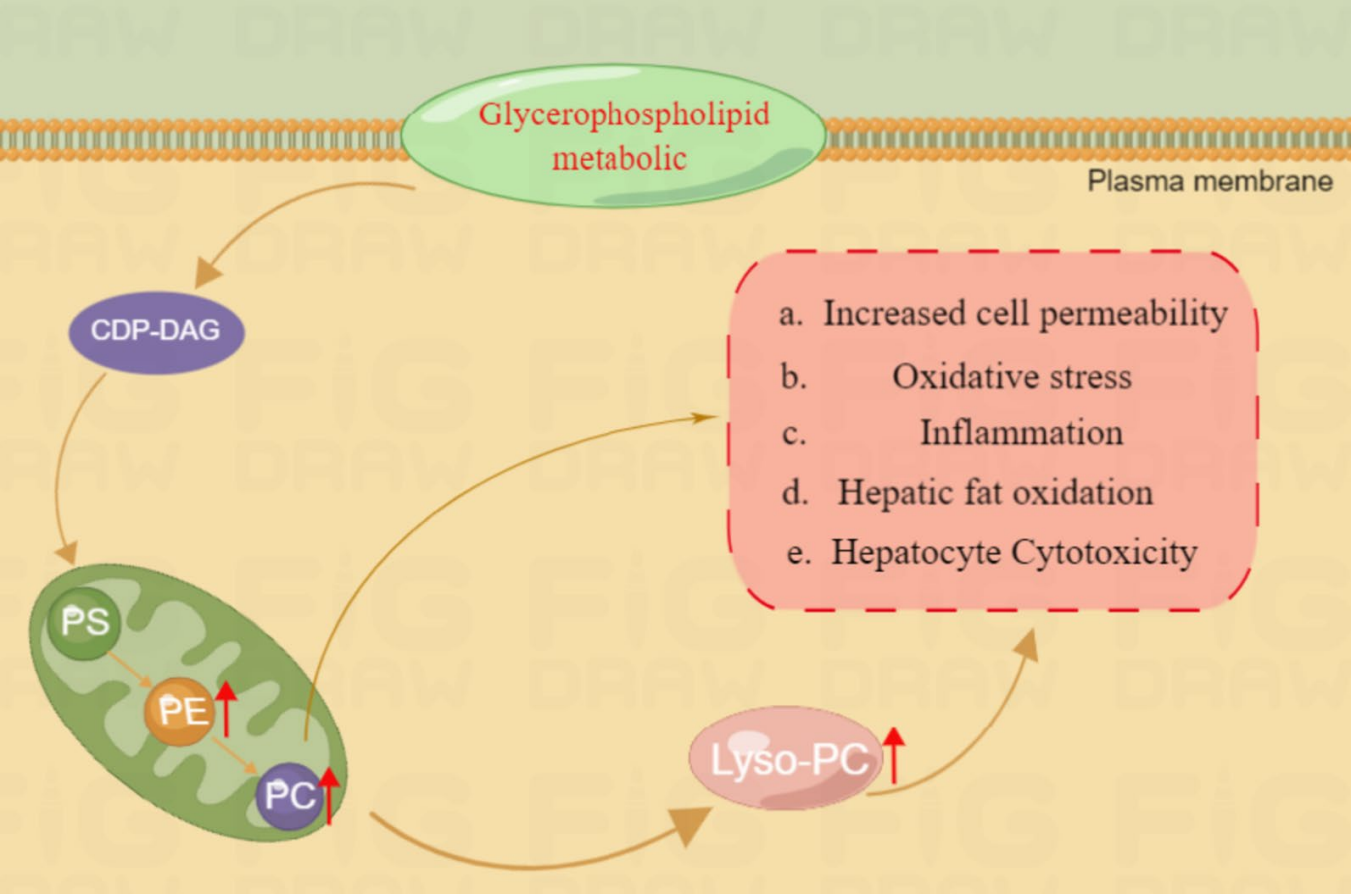
diagram of CTD affecting glycerophospholipid metabolic. PE: Phosphatidylethanolamine; PC: Phosphatidylcholine; PS: Phosphatidylserine. Lyso-PC: Lysophosphatidylcholine; CDP-DAG: CDP-diglyceride; Red upward arrows represent metabolite upregulation

PC and lyso-PC are a class of substances derived from glycerophospholipids, which play important roles in cell membrane repair and cell homeostasis. And PC is also an important lipid carrier in plasma, mainly responsible for transporting fatty acids in plasma [27]. Upregulation of lyso-PC levels has been shown to activate cholesterol biosynthesis, leading to hepatic lipotoxicity. Conversely, downregulation of lyso-PC levels could also accelerate the hepatic fatty acid oxidation process, inducing hepatic oxidative stress mechanisms, enhancing the inflammatory response and producing cytotoxicity [28]. Furthermore, a clinical study found that lyso-PC was significantly increased in the liver of NAFLD patients. Similarly, PC and lyso-PC levels have been reported to be elevated in fatty liver, further suggesting that high PC and lyso-PC levels could induce lipotoxicity, leading to cell apoptosis and causing hepatocyte damage [29]. In the present study, PC and LPC were significantly up-regulated, leading to the disorder of glycerophospholipid metabolite pathway. The above evidence substantiates that hepatic lipid imbalance and lipotoxicity could be a result from increased PC and lyso-PC levels. Concomitantly, lipid accumulation in the liver may induce apoptosis and liver injury by altering cell membrane permeability, stimulating inflammatory responses, and activating oxidative stress mechanisms to produce liver injury (Fig. 16).

#### Glyceride metabolic pathway

**Fig. 17:**
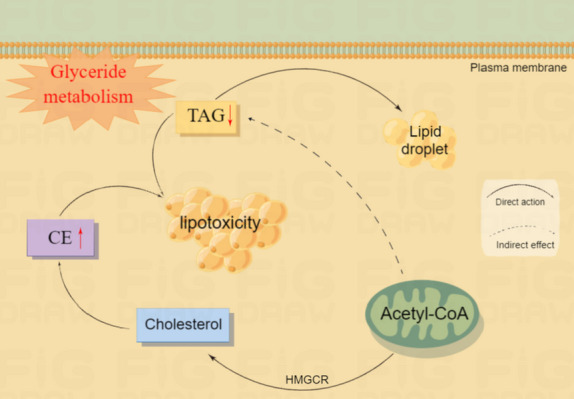
diagram of CTD affecting glyceride metabolism. CE: Cholesterol ester; TAG: Triglycerides; Red upward arrows represent metabolite upregulation; Red down arrows represent metabolite downregulation

Glycerolipids are another abundant group of plasma lipids, which contain mainly DAGs and TAGs, and a lesser amount of cholesterol and CE [30, 31]. Hepatocytes synthesize TAG mainly via the glycerol diester pathway and liver triglyceride levels are closely related to CE levels [31, 32]. Studies have shown that increased CE in the liver may compete with TAG for hydrolysis and lead to decreased TAG secretion, thereby increasing lipid storage in the liver and causing severe hepatic steatosis [33]. At the same time, hepatocytes do not store TAG but rather assemble it into very low-density lipoproteins, which are transported into the bloodstream and transferred to other tissues where they play their corresponding roles. It has been reported that Caihu Saponin leads to lipid metabolism dysfunction by increasing the secretion of TAG and cholesterol in mice, leading to lipid homeostasis imbalance and liver damage [34]. In the present study, serum TAG(16:2/18:2/18:2), TAG(18:0/18:0/20:0), TAG(13:1/19:0/19:0), TAG(18:0/18:0/22:0) and 24 other TAG metabolites were significantly decreased and CE(16:0), and CE(18:0) were increased, suggesting that CTD-caused liver injury may be due to increased CE inhibiting TAG hydrolysis, resulting in lower serum TAG levels, which in turn disturbed glyceride metabolism leading to liver injury (Fig. 17).

#### Glycosylphosphatidylinositol (GPI)-anchored biosynthesis and metabolism

The synthesis of glycoproteins and glycolipids is one of the main functions of the liver, and GPI anchoring modification is one of the common post-translational modifications of eukaryotic cell membrane surface proteins. As one of the main components of cell membranes, PE plays an important role in maintaining cell stability. The increase of PE causes a disbalance in the PC/PE ratio, resulting in aggravated membrane oxidative stress [35]. GPIs anchor proteins outside the cytoplasmic membrane through glycosylation, resulting in abnormal glycosylation that affects the structure and function of the liver [36]. In the present study, the lipid metabolites of PE (14:0e/21:2) were significantly increased after CTD intervention, suggesting that CTD may induce impairment in cell membrane function by upregulating PE levels, further inducing oxidative stress and causing liver damage.

### Clinical application strategies of CTD

As a double-edged sword, how to utilize the reduction of toxicity and enhancement of efficacy of CTD to better apply it to the clinic is an important direction for future research. In this study, a 58 serum lipid metabolic markers of CTD-induced liver injury in mice were found, which could characterize the liver injury induced by CTD. And these liver injury markers in serum could been monitored to dynamically understand the potential liver injury during the use of CTD, which could provide scientific basis for clinical avoidance and intervention of liver injury of CTD at an early stage.

## Conclusion

In conclusion, a total of 58 lipid differential metabolites, including PC, LPC and TAG were disturbed by CTD, and glycerophospholipids and glycerolipids were responsible for to play an important role in CTD-induced liver injury. The present will help to further understand the underlying mechanisms of action, expand the clinical application of CTD and provide a foothold for future studies.

## Data Availability

The corresponding author (Xiaofei, Lixiaofei@zmu.edu.cn; Li Xiaomei, doctorxmm@126.com; Zhang Jianyong, zhangjianyong2006@126.com.) will provide the datasets produced and/or analysed during the current work upon reasonable request.

## References

[1] Jiang CHL, Yuan Y, Li JD (2018). Chinese Law & Government》 standard for citation of Chinese names and latin scientific names of original species of animal medicinal materials. Sci China.

[2] Xiaocui W, Yanning W, Yang N (2022). Analysis of the medication rules of prescriptions containing cantharidin in《The Dictionary of Traditional Chinese Medicine》. Guangming Traditl Chinese Med.

[3] Ren Y (2016). Cantharidin induces G2/M arrest and triggers apoptosis in renal cell carcinoma. Mol Med Rep.

[4] Zhu M (2020). Cantharidin treatment inhibits hepatocellular carcinoma development by regulating the JAK2/STAT3 and PI3K/Akt pathways in an EphB4-dependent manner. Pharmacol Res.

[5] Ma Q (2018). Unique responses of hepatocellular carcinoma and cholangiocarcinoma cell lines toward cantharidin and norcantharidin. J Cancer.

[6] Liu F (2020). Cantharidin-induced LO2 cell autophagy and apoptosis via endoplasmic reticulum stress pathway in vitro. J Appl Toxicol.

[7] Zhu SS (2019). UPLC-Q-TOF/MS based metabolomics approach to study the hepatotoxicity of cantharidin on mice. Chem Res Toxicol.

[8] Liu F (2020). Hepatoxicity mechanism of cantharidin-induced liver LO2 cells by LC-MS metabolomics combined traditional approaches. Toxicol Lett.

[9] Ming YN (2017). Liquid chromatography mass spectrometry-based profiling of phosphatidylcholine and phosphatidylethanolamine in the plasma and liver of acetaminophen-induced liver injured mice. Lipids Health Dis.

[10] Tang K (2020). Prevention of nonalcoholic hepatic steatosis by shenling Baizhu Powder: involvement of adiponectin-induced inhibition of hepatic SREBP-1c. Oxid Med Cell Longev.

[11] Malinska H (2019). Beneficial effects of troxerutin on metabolic disorders in non-obese model of metabolic syndrome. PLoS ONE.

[12] Lin Y (2021). Investigation of the idiosyncratic hepatotoxicity of Polygonum multiflorum Thunb. through metabolomics using GC-MS. BMC Complement Med Ther.

[13] He T (2021). Integrating non-targeted metabolomics and toxicology networks to study the mechanism of Esculentoside A-induced hepatotoxicity in rats. J Biochem Mol Toxicol.

[14] Wang Y, Feng F (2019). Evaluation of the hepatotoxicity of the Zhi-Zi-Hou-Po decoction by combining UPLC-Q-exactive-MS-based metabolomics and HPLC-MS/MS-based geniposide tissue distribution. Molecules.

[15] Zhang J (2020). Study on the mechanism of cantharidin-induced hepatotoxicity in rat using serum and liver metabolomics combined with conventional pathology methods. J Appl Toxicol.

[16] Liu X, Xu G (2018). Recent advances in using mass spectrometry for mitochondrial metabolomics and lipidomics - A review. Anal Chim Acta.

[17] Wang R (2020). Integration of lipidomics and metabolomics for in-depth understanding of cellular mechanism and disease progression. J Genet Genomics.

[18] Xu S (2019). lipidomic profiling reveals disruption of lipid metabolism in valproic acid-induced hepatotoxicity. Front Pharmacol.

[19] Huang W (2017). Protective Effect of Flavonoids from Ziziphus jujuba cv. Jinsixiaozao against acetaminophen-induced liver injury by inhibiting oxidative stress and inflammation in mice. Molecules.

[20] Wu L, Parhofer KG (2014). Diabetic dyslipidemia. Metabolism.

[21] Chang H (2017). Identification of key metabolic changes during liver fibrosis progression in rats using a urine and serum metabolomics approach. Sci Rep.

[22] Wang S (2020). Effect of (R)-salbutamol on the switch of phenotype and metabolic pattern in LPS-induced macrophage cells. J Cell Mol Med.

[23] Hernandez-Alvarez MI (2019). Deficient endoplasmic reticulum-mitochondrial phosphatidylserine transfer causes liver disease. Cell.

[24] Wang X (2022). Salidroside, a phenyl ethanol glycoside from Rhodiola crenulata, orchestrates hypoxic mitochondrial dynamics homeostasis by stimulating Sirt1/p53/Drp1 signaling. J Ethnopharmacol.

[25] Hou Y (2023). Salidroside intensifies mitochondrial function of CoCl(2)-damaged HT22 cells by stimulating PI3K-AKT-MAPK signaling pathway. Phytomedicine.

[26] Shi Q (2021). Transcriptome and lipid metabolomics-based discovery: glycyrrhizic acid alleviates tripterygium glycoside tablet-induced acute liver injury by regulating the activities of cyp and the metabolism of phosphoglycerides. Front Pharmacol.

[27] Li C (2022). Lipidomics indicates the hepatotoxicity effects of EtOAc extract of rhizoma paridis. Front Pharmacol.

[28] Huang W, Xie P, Cai Z (2020). Lipid metabolism disorders contribute to hepatotoxicity of triclosan in mice. J Hazard Mater.

[29] Han MS (2008). Lysophosphatidylcholine as a death effector in the lipoapoptosis of hepatocytes. J Lipid Res.

[30] Yan F (2017). Identification of the lipid biomarkers from plasma in idiopathic pulmonary fibrosis by Lipidomics. BMC Pulm Med.

[31] Loh K (2019). Inhibition of adenosine monophosphate-activated protein kinase-3-Hydroxy-3-methylglutaryl coenzyme a reductase signaling leads to hypercholesterolemia and promotes hepatic steatosis and insulin resistance. Hepatol Commun.

[32] Liang YH (2016). Serum metabonomics study of the hepatoprotective effect of Corydalis saxicola Bunting on carbon tetrachloride-induced acute hepatotoxicity in rats by (1)H NMR analysis. J Pharm Biomed Anal.

[33] Alger HM (2010). Inhibition of acyl-coenzyme A:cholesterol acyltransferase 2 (ACAT2) prevents dietary cholesterol-associated steatosis by enhancing hepatic triglyceride mobilization. J Biol Chem.

[34] Li X (2017). Saikosaponins induced hepatotoxicity in mice via lipid metabolism dysregulation and oxidative stress: a proteomic study. BMC Complement Altern Med.

[35] Li Z (2006). The ratio of phosphatidylcholine to phosphatidylethanolamine influences membrane integrity and steatohepatitis. Cell Metab.

[36] Lipinski P (2021). Liver involvement in congenital disorders of glycosylation and deglycosylation. Front Pediatr.

